# Impact on radiological practice of active guideline implementation of musculoskeletal guideline, as measured over a 12-month period

**DOI:** 10.1177/2058460120988171

**Published:** 2021-03-17

**Authors:** Ann M Gransjøen, Kjetil Thorsen, Kristin B Lysdahl, Siri Wiig, Bjørn M Hofmann

**Affiliations:** 1 Department of Health Sciences, Norwegian University of Science and Technology (NTNU), Gjøvik, Norway; 2 The Norwegian Air Ambulance Foundation, Oslo, Norway; 3 Department of Optometry, Radiography and Lighting Design, University of South-Eastern Norway, Kongsberg, Norway; 4 SHARE-Centre for Resilience in Healthcare, University of Stavanger, Stavanger, Norway; 5 Center for Medical Ethics, University of Oslo, Blindern, Oslo

**Keywords:** Musculoskeletal radiology, x-ray, Computed Tomography, Magnetic Resonance Imaging, ultrasound, radiological guidelines, guideline implementation, interrupted time series

## Abstract

**Background:**

An ever-increasing technological development in the field of radiology urges a need for guidelines to provide predictable and just health services. A musculoskeletal guideline was developed in Norway in 2014, without active implementation.

**Purpose:**

To investigate the impact of active guideline implementation on the use of musculoskeletal diagnostic imaging most frequently encountered in general practice (pain in the neck, shoulders, lower back, and knees).

**Material and Methods:**

The total number of outpatient radiological examinations across modalities registered at the Norwegian Health Economics Administration between January 2013 and February 2019 was assessed using an interrupted time series design.

**Results:**

A 12% reduction in the total examination of Magnetic Resonance Imaging shoulder and knee, and x-ray lower back and shoulder was found at a significant level (*p* = 0.05). Stratified analysis (Magnetic Resonance Imaging examination as one group and x-ray examinations as the other) showed that this reduction mainly was due to the reduction in the use of Magnetic Resonance Imaging examinations (shoulder and knee) which was reduced by 24% at a significant level (*p* = 0.002), while x-ray examinations had no significant level change (*p* = 0.71). No other statistically significant changes were found.

**Conclusion:**

The impact of the implementation on the use of imaging of the neck, shoulder, lower back, and knee is uncertain. Significant reductions were demonstrated in the use of some examinations in the intervention county, but similar effects were not seen when including a control group in the analysis. This indicates a diffusion of the implementation, or other interventions or events that affected both counties and occurred in the intervention period.

## Introduction

Radiology has seen an immense technological advancement in the last decades. These advances have led to new and more information-heavy modalities such as Computed Tomography (CT), Magnetic Resonance Imaging (MRI), ultrasound (US), Positron Emission Tomography, and others. These advances have also led to a stronger need for guidelines, as the complexity of the field has increased, new options for diagnostic imaging has inflated,^1^ the unwarranted variations in use of imaging became evident, and the awareness of unnecessary imaging increased.^2–4^

Guidelines have mostly been developed to target the referrer’s behavior, such as the American College of Radiologists Appropriateness Criteria,^5^ and the iRefer developed by the Royal College of Radiologists.^6^ In Norway, a guideline for diagnostic imaging for non-traumatic musculoskeletal diseases was finalized in 2014 as a response to increasing number of options of diagnostic imaging for a large patient group (19% of all consultations in general practice).^7^ A significant number of these patients are referred to diagnostic imaging, and significant geographical variations are demonstrated in the use of this type of imaging.^8–10^ A high utilization rate of examinations with unclear benefit has been reported, e.g. MRI of the shoulder.^9^

However, the guideline developed in Norway was neither widely known nor used.^11^ This may be because the guideline was implemented by mail and online publishing, and not tailored toward the target group.^11^ For this evaluation, an implementation strategy tailored to the target groups of the Norwegian musculoskeletal guideline was developed.^12^ This was a multifaceted strategy, including educational meetings and videos, as well as a tailored short version of the guideline including recommendations for diagnostic imaging of the neck, shoulder, lower back and knee, and online publishing in appropriate media.^12^

The hypothesis made for this study was that the implementation of the musculoskeletal guideline would lead to a reduction in musculoskeletal imaging, due to the assumption of there being a relatively high rate of unwarranted imaging in this specific field of radiology. We here define unwarranted imaging as examinations where the results, negative or otherwise, does not lead to a change in the patient handling (diagnostics or treatment), or when leading to a change in patient handling resulting in a worse outcome.^6^

The aim of the current study was to investigate the effect of this implementation strategy on the use of diagnostic imaging of the musculoskeletal system for the four body parts focused on in the implementation in total, and for some specific examinations related to unwarranted examinations by using a time series analysis. The research question for this study was as follows:

*What is the outcome of active guideline implementation on radiological examinations performed in a Norwegian county, measured over a 12-month period?*

## Material and Methods

An interrupted time series (ITS) design was used in this study, which is a quasi-experimental design that can be used to estimate intervention effect using longitudinal data,^13^ where historical data are used to establish underlying trends, interrupted by an intervention at a fixed-point time.^14^ The data were modeled by segmented linear regression with a discontinuity at the intervention period. Then the effect of the intervention is estimated by performing hypothesis tests on the change of the level and the slope of the regression model across the intervention period.^15^ ITS was used in this study since it is the strongest quasi-experimental research design when randomization is not possible.^16^ In addition, it can account for natural trends and seasonal variations, which is expected in the current study.

### Preparation of the data

The data in this study consisted of the total number of the selected radiological examinations relating to the musculoskeletal system registered at the Norwegian Health Economics Administration (HELFO) between 1 January 2013 and 28 February 2019. This time period was chosen to provide historical data as far back as possible to establish any underlying trends. The implementation was performed from November 2017 to February 2018, which was the interruption, and the period chosen also provides data over a 12-month period after the implementation.

The data used in this study are registered as Norwegian Classification of Radiological Procedures (NCRP) codes. HELFO registers data of outpatient examinations performed at public hospitals and private institutions. Any in-patient examinations and examinations covered by insurance or paid in full by the patient are not included in this data.

The NCRP codes included were those related to examinations of the neck, shoulder, lower back, and knee. These body parts were chosen as focus for the implementation because pain from these locations are highly frequent conditions met in primary care.^17^ All modalities are included, i.e. Conventional Radiography (CR), MRI, CT, and US.

Raw data were provided by HELFO, on our request, in the form of excel sheets in separate files for the public and private institutions, as well as for the different years. The data contained information about the treatment institution, the patient’s county of residence, and type of examination. The NCRP codes contained further information about modality (such as CR or MRI) and location (which body part was examined, such as lower back) resulting in the examination code with code explanation. The examination code was the most detailed level and indicated the specific examination performed (e.g. MRI of the knee).^9^

The aim of the intervention was to reduce the extension of unwarranted imaging performed in the intervention county. Previous studies have identified the following specific examinations to contain a high rate of unwarranted examinations:

1. MRI shoulder^18–20^
2. x-ray shoulder^7^
3. x-ray lower back^21^
4. MRI knee^22^

The impact of the intervention on the group of examinations with a high rate of unwarranted examinations were analyzed (both as specific examinations, and stratified as the MRI examinations and the x-ray examinations), as well as stratified analysis on the subgroups examinations of the neck, shoulder, lower back, or knee. Finally, the impact of the intervention was analyzed on the total of the selected examinations (neck, shoulder, lower back, and knee).

Two counties were studied; the county where the re-implementation were held and an independent control county. This was done to control for any non-random effects that may have occurred during the intervention period. A total of 139,953 examinations were performed in the intervention county and 259,423 examinations in the control county in the specific time-period. The data were then sorted into their respective counties and sorted into the different imaged body parts (neck, shoulder, lower back, and knee), in addition to respective modalities, and finally specific procedures.

Inclusion criteria for the implementation were: target groups from the planning stage (radiologists and GP’s) from the intervention county were included in the implementation, and radiographers and the Norwegian Labour and Welfare Administration (NAV) were included as well.

Exclusion criteria for implementation: radiological personnel or GP’s who were not employed in a hospital/radiological institution, non-medical members of staff from NAV, or not from the intervention county.

Inclusion criteria for statistical analysis: examinations of the neck, shoulder, lower back, and knee since these were the focus areas of the intervention, and the specific examinations within these areas presumed to have a high proportion of unwarranted imaging.

Exclusion criteria for statistical analysis: systematic outliers or datapoints clearly violating a linear trend.

In order to adjust for population size changes over time and differences in size between intervention and control group, the number of diagnostic imaging were normalized by dividing on the population size. Only data for annual population sizes where available, thus a linear change were assumed. All data are expressed as counts per 100,000.

**Fig. 1.**
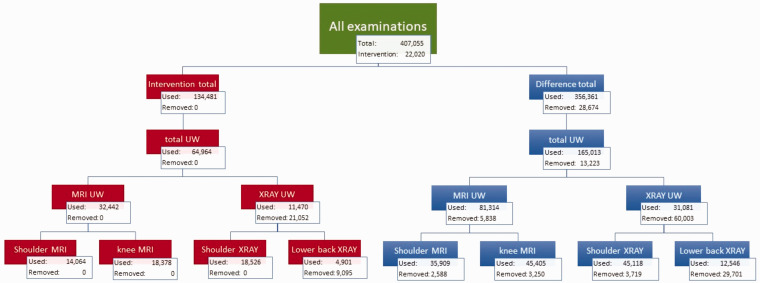
This flow chart shows the process of removing datapoints such as systemic outliers prior to the statistical analysis for imaging of the neck, shoulder, lower back, and knee. The chart shows the process both prior to analysis of the intervention county alone and analysis of the difference between the intervention and control counties. UW: Unwarranted; MRI: Magnetic Resonance Imaging.

The raw data were plotted and assessed for any linear trends, repeating patterns, and outliers. Upon visual inspection of the data, a potential trend change in the x-ray lower back time series for the intervention county was noticed. The trend change was confirmed to be significant by an ITS, and thus data before August 2016 where excluded for further analysis for this time series. For the difference time series, it was noticed systematic outliers for all the time series, thus the following five datapoints were removed from the analysis: October 2013, January 2014, December 2015, January 2016, and February 2016 (which corresponds to datapoint number 10, 13, 36, 37, and 38). See Fig. 1 for a detailed overview of the datapoints removed.

### Statistical analysis

The statistical analysis will be based on a segmented regression analysis. The following formula gives the base model

$$\begin{array}{c} f_{\text{base}}\left(t\right)=\beta_{0}+\beta_{1}\;t+\beta_{2}\;u\left(t-t_{\text{int}}\right)\; \end{array}$$

(1) $$\begin{array}{c} +\beta_{3}\;\left(t-t_{\text{int}}+1\right)u\left(t-t_{\text{int}}\right)+\eta_{t} \end{array}$$

where *t* is the timestep, $t_{\mathrm{int}}$ is the fixed timestep when post intervention starts, and *u*(*t*) is the unit step function, and $\eta_{t}$ is the noise at timestep *t*. All the noise terms will be assumed to be independent and identical normally distributed. The constants $\beta_{0},\;\beta_{1},\;\beta_{2},\;\beta_{3}$ will be estimated using linear regression. The basic model is then compared to more complex models that also include one or more dummy variables and/or harmonic terms to adjust for seasonality. The optimal model is then selected based on fit and parsimony (using R^2^ and Akaike Information Criterion for small sample size). The residuals (noise) of this model are then checked against the assumptions for independence using autocorrelation- and partial autocorrelation function plots. Normality was assessed using density plots, Quantile–Quantile plots, and the Shapiro–Wilk’s test. If the regression assumptions where not met, the noise term was further modeled by potential Seasonal Autoregressive Integrated Moving Average (SARIMA) processes.^23^ The most parsimonial model that met the regression assumptions where then selected as the final model.

The effect of the intervention was then estimated by a hypothesis test on the regression coefficients for the level change and slope ($\beta_{2},\;\text{and}\;\beta_{3}$). A *p*-value of 0.05 or less was considered statistically significant. A summary of the final models and results are given in Table 1. All the analyses were performed in R 3.6.0.

**Table 1.** Summary of the analysis performed, including adjustments made to the base model (equation (1)), and the results of the analysis (level change and trend change).

	Model			Effect of intervention			
	Signal	Noise (SARIMA)	Removed	Level change		Slope change	
Time series	covariates	(p,d,q,P,D,Q,m)	points (#)	Estimation	p-value	Estimation	p-value
Oppland							
Shoulder MRI	S_7_, S_12_	(0,0,0,0,0,1,7)	–	–16.8	0.095	0.053	0.965
Shoulder x-ray	S_7_,H(2,12)	(0,0,0,1,0,0,11)	–	–5.0	0.622	–0.419	0.731
Lower back x-ray	S_7_, S_12_	–	1–43	–4.6	0.602	0.600	0.557
Knee MRI	S_7_,S_12_,H(2,12)	(0,0,0,0,0,2,12)	–	–20.8	0.101	–0.108	0.943
Total	S_7_, S_12_	–	–	–127.8	0.064	3.191	0.709
MRI UW	S_7_, S_12_	(0,0,0,0,0,2,4)	–	–70.5	0.002	3.130	0.304
x-ray UW	S_7_	–	1–43	7.7	0.711	0.857	0.709
Total UW	S_7_, S_12_	–	–	–62.8	0.049	1.363	0.731
Difference							
Shoulder MRI	Base	–	10,13,36–38	–13.9	0.278	0.076	0.962
Shoulder x-ray	Base	–	10,13,36–38	20.6	0.105	1.524	0.330
Lower back x-ray	Base	–	1–43	–4.7	0.629	–0.006	0.995
Knee MRI	Base	(1,0,0,0,0,1,13)	10,13,36–38	–10.0	0.566	–0.104	0.961
Total	Base	(1,0,0,0,0,0,0)	10,13,36–38	30.7	0.728	1.423	0.900
MRI UW	Base	(2,0,1,0,0,0,0)	10,13,36–38	0.0	1.000	–0.943	0.803
x-ray UW	Base	–	1–43	15.6	0.301	1.194	0.472
Total UW	Base	(0,0,5,0,0,0,0)	10,13,36–38	–14.1	0.759	3.623	0.545

Notes: Difference refers to the difference in amount of diagnostic imaging performed between the intervention and the control county. UW refers to examinations most likely containing a high rate of unwarranted imaging.

UW: Unwarranted; ${\;S}_{i}$: seasonal term for month number i; H(2,12): a harmonic term of order 2 and period 12; MRI: Magnetic Resonance Imaging.

The study has ethical approval from the Norwegian Social Science Data Services (Ref. 48267, 6 May 2016).

## Results

**Fig. 2.**
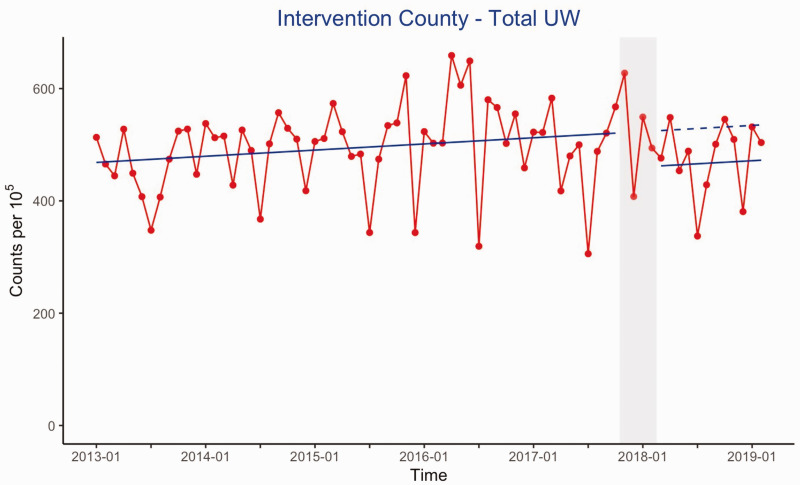
Plot showing the total use of the examinations most likely to contain a high rate of unwarranted imaging (MRI of the shoulder and knee, and x-ray of the lower back and shoulder). The *y*-axis shows the number of examinations performed per 10^5^ inhabitants in the intervention county, and the *x*-axis shows the timeline in years. The blue line shows the estimated averages trend, and the stippled blue line shows the counterfactual trend (the trend if the intervention had not been performed). The intervention period is shown in gray. This plot shows a significant reduction in level (*p* = 0.05) in the use of these imaging procedures. UW: unwarranted.

We found a statistically significant level change for the intervention county for all four examinations most likely to contain a high degree of unwarranted examinations (MRI shoulder and knee, and x-ray lower back and shoulder) analyzed together. The level change seen here equates a reduction in the use of these examinations of 62.8 examinations per 10^5^, per month, which is an average reduction of 11.9% the first year after the intervention (*p* = 0.05). See Fig. 2 for an overview of this change. Taking into account the positive slope change found in the analysis for the four specific examinations, this indicates a temporary effect, but this is not statistically significant.

**Fig. 3.**
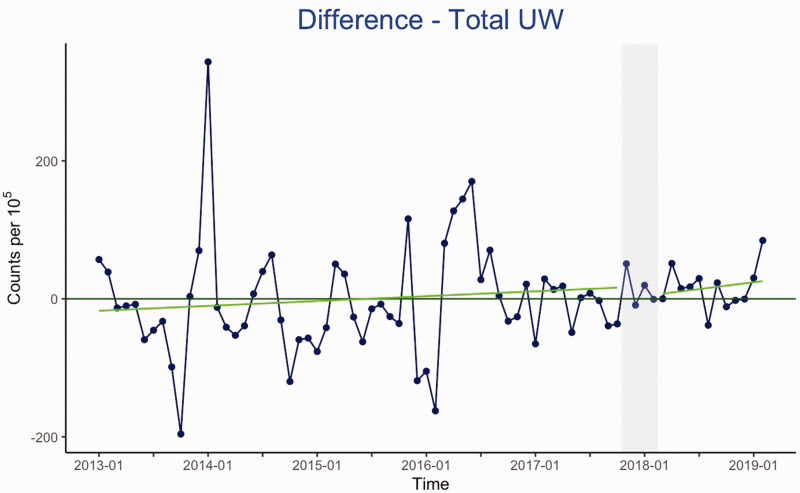
This plot shows the difference in the total use of the examinations most likely to contain a high rate of unwarranted imaging (MRI of the shoulder and knee, and x-ray of the lower back and shoulder) between the intervention and control county. Plot showing the total use of the examinations most likely to contain a high rate of unwarranted imaging (MRI of the shoulder and knee, and x-ray of the lower back and shoulder). The *y*-axis shows the number of examinations performed per 10^5^ inhabitants in both counties, and the *x*-axis shows the timeline in years. The intervention period is shown in gray. The numbers are centered at 0 for easier interpretation, and the green line shows the estimated averaged trend line. No significant effect of the intervention is shown here. UW: unwarranted.

A reduction of 70.5 examinations per 10^5^ per month, which corresponds to an average reduction of 23.4% per month the forts year after the intervention (*p* = 0.002), was found for the use of MRI examinations previously shown to contain a high rate of unwarranted examinations (MRI shoulder and knee) for the intervention county when analyzed together. These significant effects were not seen in the comparison between the intervention and control county (see figure 3).

For the analysis of the specific examinations alone, the four subgroups chosen (neck, shoulder, lower back, and knee) and the subgroups combined (total) yielded no statistically significant results. This was also the case for the analysis of the difference in use of imaging between the intervention and control county. For an overview of all the results, see Table 1.

## Discussion

The hypothesis made of this study was that a successful implementation of the musculoskeletal guideline would lead to a reduction in musculoskeletal imaging, due to a high rate of unwarranted imaging in this specific field of radiology. Since we cannot measure the portion of unwarranted imaging directly in this study, we had to assume that a reduction in unwarranted imaging would lead to a detectable decrease in the use of musculoskeletal imaging. On the other hand, the total use of diagnostic imaging would remain stable, if more warranted procedures are performed. However, the reduction found in the total use of MRI shoulder and knee, and x-ray of the lower back and shoulder combined indicate a reduction in unwarranted imaging, since these are all examinations that are considered to have a high proportion of unwarranted imaging.

Previous implementation efforts in other contexts have shown that guideline implementation is complex, and lasting change is challenging to achieve. Moreover, there is no quick fix as no type of implementation has shown to be the most effective.^24^ Nonetheless, more active approaches to guideline implementation such as educational meetings and outreach,^25^ and audit and feedback^26^ may give approximately 20% reduction passive approaches such as postal dissemination where the effect range from none^27,28^ up to 10% difference between intervention and control group.^29^

In the current study, the intervention had a significant reduction in the four selected unwarranted examinations (MRI shoulder and knee, and x-ray lower back and shoulder).

This is most likely due to the reduction in MRI examinations, since the x-ray examinations increased after the intervention. This reduction may be due to a larger potential for change in these examinations, especially for MRI of the shoulder, where approximately half have been found to be unwarranted.^18–20^ The recent focus on reducing surgeries of both the shoulder and the knee from the government may also have had an effect on the use of these examinations, commonly used as an evaluation tool prior to surgery.^30^ There has also been a higher focus on unwarranted tests and examinations in general through the launch of the choosing wisely campaign in Norway,^31^ which may make referrers and radiological personnel more aware of this phenomenon.

The fact that we found no statistical significant change in the use of the x-ray examinations may be due to an already existing downward trend in the use of these examinations. Another factor that can have contributed to this finding may be that the changes made on the basis of the implementation did not lead to a reduction in the use of these examinations, but rather changes like the referrer choosing to postpone a referral for imaging. This means that the imaging is still performed; however, the imaging may be more justified than it would have been if it was performed at an earlier stage.^32^

However, the significant effects were not reproduced when using the base model for analyzing the difference between the intervention and control county. This indicates that there has been something affecting the use of these examinations in both the intervention and the control county. In addition to the just-mentioned government initiatives, this may be due to the web-based dissemination through publishing the guideline on the Norwegian Electronic Medical manual. This online resource used by most Norwegian GPs could not be contained to the intervention county only, and since this is the measure most likely to lead to this effect, since the government initiatives where started in 2019 and 2018, respectively. Despite web-based dissemination of guidelines being found to lead to significant change in use of imaging elsewhere,^33^ continued geographical variations,^9,10^ and lack of knowledge of the guideline^11^ after earlier attempts of online dissemination indicate that this may not be the case in Norway. In addition to this, there may have been efforts performed that we are not aware of.

The limited effect of the implementation in the current study compared to previous studies may be related to differences in the implementation content. For example, the implementation may have had a greater effect if individual feedback on referral rates had been provided to the participants, which has shown potential for reducing referrals for diagnostic imaging in previous studies.^34,35^ The participants in our implementation missed, and would like more feedback. However, the feedback missed by the GPs in our study was not referral rated, but rather the quality and justification of their referrals.^32^ Reminders have also proven to be effective means to change behavior,^26,36^ which potentially could have improved the effect of the implementation. However, it was chosen not to include reminders on the basis of the interviews with GPs and radiologists, as well as discussions with a practice coordinator, where the emphasis was mainly on easy accessibility of guidelines. In other words, the possible reasons that these approaches achieved greater effect than we did could be more concrete guiding approaches and individual attention. Even though the target groups were involved in the development of the implementation strategy to some degree, further involvement of the target groups could have improved the implementation strategy by increased tailoring toward the users, which could have improved the effect of the implementation. This may also be the case for the development of the guideline itself, where further involvement of the target groups could have improved the composition of the guideline, tailoring it toward the needs of the target groups and thereby making it easier to use in day-to-day life.

The differences in effect may also be explained in terms of differences in context. For example, it can be assumed that the distribution of power in the decision-making process between the referring physician and the radiologist may differ between different countries.

The implementation delivery could also have been improved by further utilization of the Consolidated Framework for Implementation Research (CFIR),^37^ which could have led to a clearer intervention, and improving follow-up in the intervention county. This includes more extensive inclusion of the target groups in the planning stage, increased tailoring of the implementation strategy, and more appropriate style, imagery, and language used for the educational parts of the implementation.^37^ A more extensive use of CFIR may also have included closer follow-up of the participants through group debriefings, which may have further increased awareness of the guideline, and thereby facilitated guideline use.^37^ This may in turn have increased the effect of the implementation. It may also be the case that CFIR was not the most optimal framework to use, in regards of the conduction of the implementation. Other frameworks, like the Promoting Action on Research Implementation in Health Service (PARiHS) framework could have drastically changed the implementation and target groups included. The PARiHS framework have a higher focus on research implementation being an organizational issue rather than an individual issue, and strategies consisting of a range of intervention that address the need for education, audit, and the management of change.^38^ However, CFIR was chosen because it is more comprehensive.^32^

Other factors related to the conduction of the implementation that may have influenced the outcome could be the fact that the educational meetings were not information that the participants sought out as an answer for a perceived problem. It was offered to a representative of the participating municipality, which may have led to the information not being perceived as equally interesting for all the participants, since some participants viewed the implementation more as a confirmation that their existing practice was correct, rather than as an opportunity to improve.^32^ The frequency (or dose) of the implementation may also influence the outcome. In the current study, a repeat of the educational meetings, or other means of repeating the information could imply greater effect on the use of unwarranted imaging, and thereby a significant change in the difference in use of unwarranted imaging between the intervention and control counties. This lack of change in difference between counties may also be explained by other factors than the frequency of the implementation, such as the implementation not being comprehensive enough, or poor coverage of the target groups.

Guideline implementation in general is complex, where many factors need to be taken into consideration to accomplish the desired effect, such as the quality of the evidence, readiness for change, and stakeholder involvement. Several types of intervention may be needed to facilitate guideline use, since a single intervention may not address all the factors needed for change to be achieved. There is no evidence as to which type of implementation is the most effective; however, it does seem that the more active approaches of greater duration and frequency are consistently more successful in achieving change than single interventions, or one-off implementation efforts.^39^ In the current study, a tailored implementation strategy was applied; however, the effect was limited. Another contributing factor to the limitation of the effect may be the complexity of the referral context. The ever increasing number of guidelines may result in “guideline fatigue”^27^ and may foster a guideline-resistant context. Publication bias may also be an explaining factor, since studies reporting positive results have historically been more likely to be published than studies with null-results.^40^

Limitations of this study are first, a relatively small sample of observations after the implementation, which may affect the result. In addition to this, 42 datapoints were removed for the analysis of the use of x-ray of the lower back, due to a significant level change Medio 2016, unrelated to the implementation. This may be caused by other and unacknowledged efforts to reduce the number of unwarranted imaging of the lower back, or increased media attention to excessive x-ray imaging of the lower back at the time. The removal of these datapoints led to a relatively small sample of observations for both the period before and after the intervention, which may influence the findings.

However, the ITS design is very robust, where other implementation efforts at the same time are what would have the most negative effect on the reliability of the results, rather than the number of datapoints. It cannot be ruled out that the observed effect in this study is only in part related to the implementation. As previously mentioned, other efforts performed in the same period as the implementation that may explain the lack of difference between the counties.

The study is also based solely on the number of examinations, without information regarding the justification of the examinations. First, some of the examinations may not have musculoskeletal indications, and second we have no exact number of unwarranted imaging. Hence, the number of examinations is only an indicator of unwarranted examinations. Given abundant waiting lists, there may be more indicated examinations and less non-indicated examinations resulting from the intervention.

Another limitation of the study is that it is a retrospective design. In this case, we had to rely on the accurate coding for the examinations, and we did not have control of any other event possibly influencing the intervention or the control group, as already mentioned. Finally, the intervention performed was relatively small, in terms of its duration and the fact that the meetings were not repeated, which can have had an effect on the results.

In conclusion, the impact of a multifaceted implementation of musculoskeletal referral guidelines on the use of diagnostic imaging of the neck, shoulder, lower back, and knee is uncertain. There was found a significant reduction in the use of MRI examinations deemed most likely to have a great portion of unwarranted imaging in the intervention county, indicating a reduction in unwarranted imaging. However, a similar effect was not found when including a control county, or other interventions or events that affected both counties and occurred in or around the intervention. Further implementation efforts should concentrate not only on tailoring the implementation toward the group, but also on several other factors. Such as adequate covering of the target group(s), ensuring that the users are involved in the entire implementation process from defining the problem, to designing and delivering the implementation, as well as ensuring sufficient uptake and follow-up.
